# Quantification of Phase-Amplitude Coupling in Neuronal Oscillations: Comparison of Phase-Locking Value, Mean Vector Length, Modulation Index, and Generalized-Linear-Modeling-Cross-Frequency-Coupling

**DOI:** 10.3389/fnins.2019.00573

**Published:** 2019-06-07

**Authors:** Mareike J. Hülsemann, Ewald Naumann, Björn Rasch

**Affiliations:** ^1^Division of Cognitive Biopsychology and Methods, Department of Psychology, University of Fribourg, Fribourg, Switzerland; ^2^Department of General Psychology and Methodology, Faculty I – Psychology, University of Trier, Trier, Germany

**Keywords:** phase-amplitude cross-frequency coupling, phase-locking value, mean vector length, modulation index, GLM-CFC, simulated EEG/MEG data

## Abstract

Phase-amplitude coupling is a promising construct to study cognitive processes in electroencephalography (EEG) and magnetencephalography (MEG). Due to the novelty of the concept, various measures are used in the literature to calculate phase-amplitude coupling. Here, performance of the three most widely used phase-amplitude coupling measures – phase-locking value (PLV), mean vector length (MVL), and modulation index (MI) – and of the generalized linear modeling cross-frequency coupling (GLM-CFC) method is thoroughly compared with the help of simulated data. We combine advantages of previous reviews and use a realistic data simulation, examine moderators and provide inferential statistics for the comparison of all four indices of phase-amplitude coupling. Our analyses show that all four indices successfully differentiate coupling strength and coupling width when monophasic coupling is present. While the MVL was most sensitive to modulations in coupling strengths and width, only the MI and GLM-CFC can detect biphasic coupling. Coupling values of all four indices were influenced by moderators including data length, signal-to-noise-ratio, and sampling rate when approaching Nyquist frequencies. The MI was most robust against confounding influences of these moderators. Based on our analyses, we recommend the MI for noisy and short data epochs with unknown forms of coupling. For high quality and long data epochs with monophasic coupling and a high signal-to-noise ratio, the use of the MVL is recommended. Ideally, both indices are reported simultaneously for one data set.

## Introduction

Phase-amplitude coupling is a promising measure to study cognitive processes (Jensen and Lisman, 1998; Jensen, 2006; Lisman and Jensen, 2013; Vosskuhl et al., 2015). There is no convention yet of how to calculate phase-amplitude coupling, but instead much heterogeneity of phase-amplitude calculation methods used in the literature. Most of these are reasonable measures from a theoretical point of view. To provide empirical evidence for choosing one of these measures over another, this work thoroughly compares the performance of the three most widely used phase-amplitude coupling measures with the help of simulated EEG data. The measures are the phase-locking value (PLV) as used in Mormann et al. (2005) (first introduced by Vanhatalo et al., 2004), mean vector length (MVL) by Canolty et al. (2006), and modulation index (MI) by Tort et al. (2008). Additionally the GLM-CFC (Kramer and Eden, 2013) is examined.

From a historical viewpoint, the first amplitude modulations that have been detected are amplitude fluctuations of specific frequency bands, becoming apparent in the fast Fourier transform (FFT) of constituents of these signals (Pfurtscheller, 1976; Novak et al., 1992; Burgess and Ali, 2002). Because the FFT approach can solely reveal that the amplitude of a higher frequency oscillates at a lower frequency (characteristic of one signal), these amplitude modulations should not be misinterpreted to account for true temporal coupling between the instantaneous phase of the lower frequency and the amplitude envelope of the higher frequency (association between two signals and definition of phase-amplitude coupling). Neither the lower frequency itself nor its instantaneous phase are extracted in this approach.

Some of the most widely used phase-amplitude coupling measures today are the PLV (Mormann et al., 2005), also called synchronization index (SI) by Cohen (2008), the MVL (Canolty et al., 2006), the MI (Tort et al., 2008), the envelope-to-signal correlation (ESC) (Bruns and Eckhorn, 2004), the generalized linear modeling (GLM) method (Penny et al., 2008; Kramer and Eden, 2013), phase binning combined with analysis of variance (ANOVA) (BA) (Lakatos et al., 2005), and the weighted phase locking factor (wPLF) (Maris et al., 2011). Recent approaches (Sotero, 2016; Martínez-Cancino et al., 2019) use mutual information in order to compute phase-amplitude coupling. The computation of mutual information is sensitive to the amount of data and noise, but advantageous when handling non-linear relationships (Cohen, 2014). All of these measures use the instantaneous phase and amplitude of band-pass filtered signals to calculate a measure that represents coupling strength. However, conceptual ideas and mathematical principles differ substantially between measures.

Several of these phase-amplitude coupling measures were compared with the help of simulated and real data in previous reviews. Tort et al. (2010) executed the most extensive comparison so far, including most of the above listed measures and evaluating their performance pertaining to tolerance to noise, amplitude independence (independence from the amplitude of the amplitude-providing frequency band), sensitivity to multimodality, and sensitivity to modulation width. The MI, introduced by the same group (Tort et al., 2008), is well-rated in all aspects while, amongst others, the PLV has poor ratings in all aspects. The MVL has good ratings in some aspects (e.g., tolerance to noise), but weaknesses in others (e.g., amplitude dependence).

Penny et al. (2008) introduced the GLM method and compared it to the PLV, MVL, and envelope-to-signal correlation in respect to noise level, coupling phase, data length, sample rate, signal non-stationarity, and multimodality. They found that the methods discriminated between data simulated with and without coupling to different extents, ranging from below chance level to perfect discrimination. Performance of the measures differed under poor conditions (high noise, low sampling rate, etc.), however, all measures performed equally well under good conditions (longer epochs, less noise, etc.).

Kramer and Eden (2013) introduced a new GLM method (GLM-CFC). It proves to be valid and performs equally well as the MI. The advantages of this method are that it can be interpreted as percentage change in amplitude strength due to modulation. Additionally confidence intervals are easily computed and the measure can detect biphasic coupling. The disadvantage of this measure is an especially high computation time due to generating the design matrix for the GLM and fitting the GLM.

When Onslow et al. (2011), compared three phase-amplitude coupling measures (MVL, envelope-to-signal correlation, cross-frequency coherence), they found that “no one measure unfailingly out-performed the others” (p. 56) (Onslow et al., 2011). They concluded that each measure seems to be particularly suited for specific data conditions. MVL for example is suitable for noisy data, exploratory analyses (analyzing a broad frequency spectrum) and when the power of the amplitude providing frequency band is low.

Samiee and Baillet (2017) statistically compared the PLV, MVL, and MI especially focusing on data length effects and the accuracy of finding the contributing coupling frequencies within exploratory analyses for broad frequency ranges. Here all three measures performed equally well in accurately finding coupling frequencies. However, their results indicate that MVL estimates coupling strength most correctly and MI is most robust to noise regarding detecting the correct coupling frequencies in the aforementioned exploratory analysis. The authors show that the performance of the direct MVL (Özkurt and Schnitzler, 2011) can be significantly increased when using sophisticated methods for detecting the actual coupling frequencies for phase and amplitude in the data and that this method allows to estimate coupling strength for very short data segments (see Samiee and Baillet, 2017 for details).

The above cited reviews do not point to a single optimal measure for calculating phase-amplitude coupling. They rather show that most – but not all – of the used measures perform well and are equally affected by various confounders. Despite the availability of manifold measures, 79% of studies use the PLV adapted for phase-amplitude coupling, MVL, or MI (Hülsemann, 2016). The three measures are indeed the three most often used. Why is this the case? The PLV is derived from a long-used, phase-phase coupling measure that is easily adapted for the purpose of phase-amplitude measurement. Its familiarity in the scientific community might have promoted its application. Possibly the predominant application of MVL is due to its mathematical directness. The MI is conceptually intuitive.

The majority of reviews used very straightforward data simulation methods. Oftentimes, a sinusoidal oscillation is constructed at a lower phase-providing frequency and at a higher amplitude-providing frequency. Phase-amplitude coupling is introduced by multiplying both signals (cf. Onslow et al., 2011). Amplitude is then extracted from the so constructed signal and phase is extracted from the pure sinusoidal oscillation of the lower frequency. White noise is added to both signals. There are two disadvantages in this approach. Both sinusoidal signals reflect a plain prototype of phase-amplitude coupling, but in real neuronal data, pure sinusoidal oscillation cannot be filtered; rather, frequency bands containing different amounts of various frequencies are extracted. Second, white noise is added to the simulated data, even though it is known that not white noise but fractional Brownian noise is inherent to brain dynamics (Miller et al., 2009; He et al., 2010).

Because none of the hitherto existing reviews simultaneously meet the requirements of realistic simulation of EEG data, providing inferential statistics for comparison of the measures, investigating moderators of phase-amplitude coupling, and including the three most widely used measures (PLV, MVL, and MI), a new comparison of these methods is presented here. Additionally the GLM-CFC is included in the comparison. We aim to combine the best aspects of all previous reviews. EEG data is simulated rather realistically according to the procedure described by Kramer and Eden (2013). The influence of several moderators (multimodality, data length, sampling rate, noise level, modulation strength, and modulation width) inspired by Tort et al. (2010) is investigated. Sensitivity and specificity of the phase-amplitude coupling measures are checked according to the methods described in Onslow et al. (2011). For all these comparisons, inferential statistics are provided.

## Materials and Methods

### Simulation of EEG Data and Implementation of Phase-Amplitude Coupling

**FIGURE 1:**
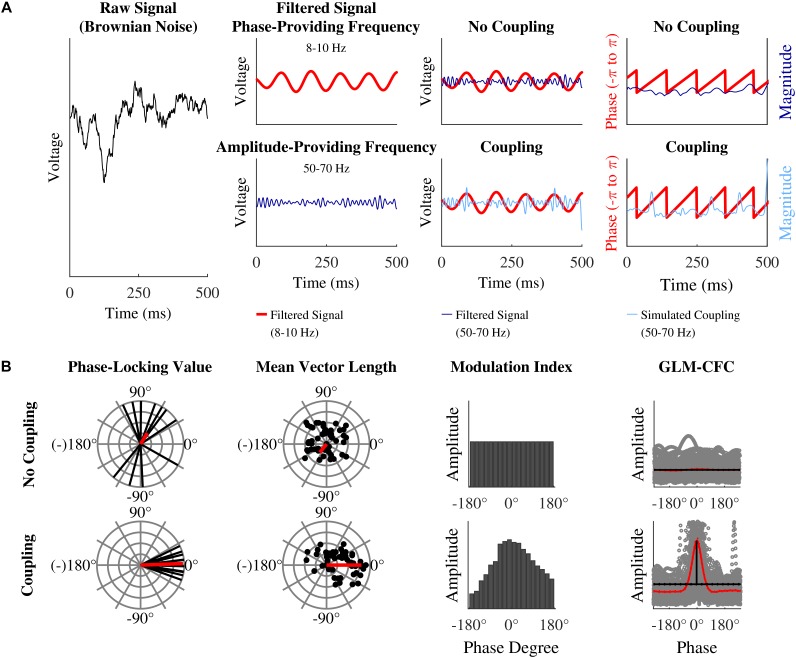
Simulation of the EEG signal and calculation of phase-amplitude coupling: **(A)** (from left to right) Brownian noise is generated. This signal is band pass filtered to extract the slow phase-providing frequency (here 8–10 Hz, red line) and the fast amplitude-providing frequency (here 50–70 Hz, dark blue line). To simulate coupling (light blue line) the amplitude-providing band pass filtered signal is multiplied with a Hanning window plus one (not depicted here), which results in stronger amplitude at the peaks of the phase-providing frequency (lower middle right panel). Before extracting phase and amplitude (most right panels) band pass filtered noise (same frequencies) is added to the filtered data (not depicted here). The simulated coupling (light blue line) amplitude is most pronounced for phases at 0°. This is not the case for the original signal (dark blue line). **(B)** Idealized depiction PLV (outer left panels), MVL (inner left panels), MI (inner right panels), and GLM-CFC (outer right panels) for a uniform distribution (upper panels) and phase-amplitude coupling (lower panels). PLV: each black line represents the phase lag between two signals at one data point. The red vector is the mean of all black vectors. The upper panel shows inconsistent, widespread phase lags. The widespread phase lags lead to a relatively short mean vector (red line). The outer left lower panel shows an example of a relative constant phase lag around 0°. A relative constant phase lag leads to a relatively long mean vector. MVL: each black dot represents one data point of the analytical signal. In case of coupling, a portion of the dots (or vectors) are especially long (reflecting strong amplitudes) at a specific narrow range of phase angles (here 0° in the lower panel). The red vector is the mean of all black vectors. It reflects coupling strength (short for no coupling – long for coupling). In case of phase-amplitude coupling it is indicating the preferred phase. MI: all possible phases are binned into 18 bins of 20° from –180 to 180°. Each bar reflects the mean amplitude of the amplitude-providing signal for the specified phase of the phase-providing frequency. This phase-amplitude plot is quantified with Shannon entropy. Shannon entropy is maximal for uniform distributions (upper panel). The Kullback–Leibler distance measures how much a given distribution (for example the one in the lower panel) deviates from the uniform distribution (depicted in the upper panel). The more phase-amplitude coupling there is in the data, the more the given phase-amplitude plot deviates from the uniform distribution and the higher the MI becomes. GLM-CFC: each circle in the scatter plot represents on data point. If there is no phase-amplitude coupling, amplitude values are rather similar across all possible phase values. In this case, a horizontal line would best model the data and the phase value would have no predictive power. If there is phase-amplitude coupling, amplitude values are specifically high at certain phase values. In this case, a curve that follows the amplitude pattern would best model the data. In case of phase-amplitude coupling, the curve (red line) differs from the horizontal line (black line) that represents no coupling. In case of no phase-amplitude coupling the curve barely differs from the null model horizontal line that represents no coupling.

A characteristic of natural EEG data is the proportionality of its frequency spectrum to a power law P(f) ∼ (1/f ^β^). Namely, the higher the frequency f, the weaker the amplitude P(f). The exponent β defines the strength of the amplitude decrease. White noise is defined by β = 0, pink noise by β = 1 and Brownian (red) noise by β = 2. Different investigations have shown that the frequency spectrum of human brain activity relates to fractional Brownian (red) noise, with 2 < β < 3 (Miller et al., 2009; He et al., 2010). Because of this, Brownian noise was generated using MATLAB code provided by Zhivomirov (2013, 2018), in order to simulate EEG data (Figure 1A).

Simulated data was then filtered at a low phase-providing frequency, from here on referred to as phase time series, with a narrow bandwidth of 2 Hz. The same data was filtered at a high amplitude-providing frequency, from here on referred to as amplitude time series, with a broad bandwidth. The exact bandwidth of the amplitude time series should depend on the frequency of the phase time series (Berman et al., 2012; Dvorak and Fenton, 2014). Because of this data was filtered such that the sidebands of the modulating frequency were always included (i.e., center frequency of amplitude-providing frequency band ± upper boundary of phase-providing frequency band).

A zero-phase Hamming-windowed sinc finite impulse response (FIR) filter implemented in EEGLAB (function *pop_eegfiltnew* contributed by A. Widmann) was used. This function automatically chooses the optimal filter order and transition band width for a precisely selectable filter bandwidth. Low frequency was set to 5–7 Hz (for *theta*-low gamma coupling) and 8–10 Hz (for *alpha*-high gamma coupling). High frequency was set to 33–47 Hz (for theta-*low gamma* coupling) and 50–70 Hz (for alpha-*high gamma* coupling). Filtering can seriously distort raw data (Widmann et al., 2015), therefore only continuous data was filtered and data segments at the beginning and end, where edge artifacts can occur, were later on discarded.

To introduce coupling, the procedure of Kramer and Eden (2013) was followed. A Hanning window plus one (i.e., each data point of the Hanning window is added with one) was multiplied with the amplitude time series. This multiplication of the Hanning window with the amplitude time series was not done continuously, but centered at either the relative maxima (peaks) or the relative maxima and minima (peaks and troughs) of the phase time series, in order to simulate monophasic and biphasic coupling, respectively. Extremum times are chosen because they are easy to detect. They relate to phase angles of 0 and 180°/-180°. Phase-amplitude coupling measures would not change if the coupling were to be introduced at another phase angle. The Hanning window itself is multiplied with the factor I to graduate the intensity of phase-amplitude coupling. To double the amplitude of the time series at the specified time I = 1.0 is chosen. I = 0.0 reflects no phase-amplitude coupling (i.e., not modulating the amplitude time series). The length of the Hanning window was also modulated to simulate different “widths” of phase-amplitude modulation. Parameters chosen for these moderators are specified below. In a final step, additional noise was added to the phase and amplitude time series. Therefore, Brownian noise of the same length was simulated, band-pass filtered at the same frequencies as the phase and amplitude time series, and added to the original phase and modulated amplitude time series, respectively. Frequency matched noise is disruptive to the modulated phase-amplitude coupling and therefore allows to check for the robustness of the phase-amplitude coupling measures.

Subsequently, phase and amplitude were extracted from the correspondent time series via Hilbert transform, using the Signal Processing Toolbox of MATLAB (The MathWorks, Inc.). Then continuous phase and amplitude time series were segmented. This was done to introduce data discontinuities, which are present in real data as well. Filtering, Hilbert transform, and phase or amplitude extraction were always conducted on continuous data, to prevent filtering or other artifacts in the later analyzed data epochs.

Each simulated data set was then modified. Data sets with a length of 42, 105, and 180 s were subsampled. This amount of data is sufficient to simulate 30 trials with a length of 400, 2500, and 5000 ms plus additional 30 s to introduce data discontinuities when segmenting the data. These parameters were chosen to mirror typical properties of event-related EEG data: (1) at least 30 trials per unique condition for which phase-amplitude coupling will be calculated (Luck, 2014), (2) trial length between 400 and 5000 ms, and (3) data discontinuities between trials. Sampling rate was set to 1000 Hz (Cohen, 2014). In addition, simulated data was resampled to 500 Hz in order to investigate the influence of sampling rate. Noise was scaled by the factor 0.9, 1.0, and 1.1 in order to simulate different signal-to-noise ratios. Scaling factor 0.9, 1.0, and 1.1 correspond to a noise signal strength of 90, 100, and 110% compared to the data signal strength. Four modulation strengths were realized: I = 0.0 for no coupling and I = 0.9, I = 1.0, and I = 1.1 for increasing coupling strength (I = 1.0 doubling the original amplitude strength). These values lie within the range of former studies (e.g., Kramer and Eden, 2013). The length of the Hanning Window ranged between 22.5 and 27.5% of one low frequency cycle to modulate different “widths” of phase-amplitude modulation. This width is equivalent to about a quarter of one cycle and therefore covers the peak (or trough) phases of that low frequency cycle. At these phases, amplitude of the higher frequency was increased. All parameters were realized for mono- and bi-phasic coupling (factor multimodality).

### Measuring Phase-Amplitude Coupling

To calculate phase-amplitude coupling, first, raw data is band-pass filtered in the frequency bands of interest. Second, the real-valued band-pass filtered signal is transformed into a complex-valued analytic signal. Finally, phase or amplitude is extracted from the complex-valued analytic signal. All these steps can essentially be implemented in MATLAB with four lines of code:

filtered_data = pop_eegfiltnew(raw_data,lower_frequency_ bound,upper_frequency_bound);

analytic_signal = hilbert(filtered_data);

phase = phase(analytic_signal);

amplitude = abs(analytic_signal).

#### Phase-Locking-Value as Used in Mormann et al. (2005)

For the calculation of the PLV, phase is extracted from the low frequency filtered analytic signal and amplitude is extracted from the high frequency filtered analytic signal. The amplitude time series is then again Hilbert transformed and phase is extracted from the “second” analytic signal. By these steps, one obtains phase angles for both time series for each data point. For each data point the phase angle of the Hilbert transformed amplitude time series is subtracted from the phase angle of the phase time series, obtaining phase angle differences.

These phase angle differences can be plotted in a polar plane as vectors of the length one with the angle representing the respective phase angle difference (Figure 1B, outer left panels). A constant phase lag between both time series indicates phase-amplitude coupling. A constant phase lag leads to vectors in the polar plane with a similar direction. Then all vectors are averaged: if they have a constant phase lag, they point into the same direction leading to a rather long mean vector. If there is a variable phase lag, the vectors are scattered around the polar plane, leading to a rather short mean vector. The length of the mean vector indicates the amount of phase-amplitude coupling (coupling strength). The direction of the vector represents the mean phase lag present between the two time series and the preferred coupling phase can be inferred from the phase lag. The PLV is calculated by the following formula:

$$\text{PLV = }|\frac{\sum_{\text{t=1}}^{\text{n}}{\text{e}}^{\text{i}({\text{θ}}_{\text{lt}}–{\text{θ}}_{\text{ut}})}}{\text{n}}|\;\;\;\;\;\;\;\;\;\;\;\;\;\;\;\;\;\;\;\;\;\;\;\;\;\;\;\left(1\right)$$

where n is the total number of data points, t is a data point, θ_lt_ is the phase angle of the lower frequency band at data point t and θ_ut_ is the phase angle of the Hilbert transformed upper frequency band amplitude time series.

The logic for this measure is as follows: if phase-amplitude coupling exists, the amplitude of the high frequency time series will oscillate at the lower frequency. In this case, extracting instantaneous phase information from this signal will return some constant phase lag to the instantaneous phase information of the low frequency band. Otherwise, inconsistent phase lags to the instantaneous phase of the lower frequency signal will be extracted, indicating no phase-amplitude coupling. A potential disadvantage of this measure is that invalid phase information will be extracted from the Hilbert transformed amplitude time series if it does not oscillate at a specific frequency. This disadvantage can be counteracted by filtering the Hilbert transformed amplitude time series in the low frequency range before extracting phase information (see Vanhatalo et al., 2004).

One should be aware that meaningful phase information can only be extracted from narrow band oscillations (Aru et al., 2015). The Hilbert transformed amplitude time series does not necessarily need to be such a narrow band oscillation.

#### Mean Vector Length by Canolty et al. (2006)

For the phase-amplitude coupling measure MVL, introduced by Canolty et al. (2006), phase is extracted from the low frequency filtered analytic signal and amplitude is extracted from the high frequency filtered analytic signal. MVL utilizes phase angle and magnitude of each complex number (i.e., each data point) of the corresponding analytic signal in a quite direct way to estimate the degree of coupling. Each complex value of the analytic time series is a vector in the polar plane. Phase-amplitude coupling is present, when the magnitude M of a fraction of all vectors is especially high at a specific phase or at a narrow range of phases (Figure 1B, inner left panels). Averaging all vectors creates a mean vector with a specific phase and length (red vector in Figure 1B). The length of this vector represents the amount of phase-amplitude coupling. The direction represents the mean phase where amplitude is strongest. When no coupling is present, all vectors cancel each other out and the mean vector will be short. Then its direction does not represent any meaningful phase. The MVL is calculated by the following formula:

$$\text{MVL = }|\frac{\sum_{\text{t=1}}^{\text{n}}{\text{a}}_{\text{t}}{\text{e}}^{{\text{iθ}}_{\text{t}}}}{\text{n}}|\;\;\;\;\;\;\;\;\;\;\;\;\;\;\;\;\;\;\;\;\;\;\;\;\;\;\;\;\;\;\;\;\;\;\;\;\left(2\right)$$

where n is the total number of data points, t is a data point, a_t_ is the amplitude at data point t and θ_t_ is the phase angle at data point t. This value cannot become negative because it represents the length of the mean vector. The length of a vector cannot be negative.

Three caveats come along with this measure: (1) the value is dependent on the general absolute amplitude of the amplitude providing frequency (independent of outliers), (2) amplitude outliers can strongly influence the MVL, and (3) phase angles are often not uniformly distributed (Cohen, 2014). All caveats are simultaneously counteracted by non-parametric permutation testing (see section “Permutation Testing”). One of the reviews cited in the introduction (Tort et al., 2010) finds faults with the MVL being amplitude dependent. However, this is only true for the raw, but not for the permuted MVL.

In the interest of completeness, it should be mentioned that Özkurt and Schnitzler (2011) proposed a direct MVL which is amplitude-normalized and ranges between 0 and 1. When applying permutation testing to both MVL and direct MVL return essentially the same values. That is, when applied along with permutation testing, both measures are exchangeable. Without permutation testing, the usage of the direct MVL is recommended because it takes care of the possible amplitude differences in raw data.

#### Modulation Index by Tort et al. (2008)

Tort et al. (2008) suggest a very different way of computing phase-amplitude coupling, which anyways is based on the same parameters of the analytic signal, amplitude magnitude and phase angle. For calculating the MI according to Tort et al. (2008), all possible phases from -180 to 180° are first binned into a freely chosen amount of bins. Tort et al. (2008) established to use 18 bins of 20° each, which many authors follow. The amount of bins can influence the results, as will be explained below. The average amplitude of the amplitude-providing frequency in each phase bin of the phase-providing frequency is computed and normalized by the following formula:

$$\text{p}(\text{j})\text{ = }\frac{\bar{\text{a}}}{\sum_{\text{k=1}}^{\text{N}}{\bar{\text{a}}}_{\text{k}}}\;\;\;\;\;\;\;\;\;\;\;\;\;\;\;\;\;\;\;\;\;\;\;\;\;\;\;\;\;\;\;\;\;\;\;\;\;\;\;\;\;\;\;\;\;\;\;\;\;\left(3\right)$$

where ā is the average amplitude of one bin, k is the running index for the bins, and N is the total amount of bins; p is a vector of N values. With the help of these calculations, one obtains the data for the phase-amplitude plot, which depicts the actual phase-amplitude coupling graphically (Figure 1B, inner right panels).

Subsequently Shannon entropy is computed; a measure that represents the inherent amount of information of a variable. If Shannon entropy is not maximal, there is redundancy and predictability in the variable. Shannon entropy is maximal, if the amplitude in each phase bin is equal (uniform distribution, Figure 1B, inner right upper panel). Shannon entropy is computed by the following formula:

$$\text{H}(\text{p})=-\sum_{\text{j=1}}^{\text{N}}\text{p}(\text{j})\text{logp}(\text{j})\;\;\;\;\;\;\;\;\;\;\;\;\;\;\;\;\;\;\left(4\right)$$

where p is the vector of normalized averaged amplitudes per phase bin and N is the total amount of bins. It does not matter which logarithm base is used if permutation testing is applied later on (Cohen, 2014). Like in Tort et al. (2008) the natural logarithm is used here. Shannon entropy is dependent on the amount of bins used and this is why the MI is likewise dependent on the number of bins. The higher the amount of bins, the larger Shannon entropy can become. Complying with the original study and most other studies, 18 bins have been employed here.

Phase-amplitude coupling is defined by a distribution that significantly deviates from the uniform distribution. Kullback–Leibler distance, a measure for the disparity of two distributions is calculated by the following formula:

KL(U,X) = logN−H(p)                            (5)

where U is the uniform distribution, X is the distribution of the data, N is the total amount of bins, and H(p) is the Shannon entropy according to Eq. 4. The uniform distribution is represented by log(N). The final raw MI is calculated by the following formula:

$$\text{MI = }\frac{\text{KL}(\text{U},\text{X})}{\text{logN}}\;\;\;\;\;\;\;\;\;\;\;\;\;\;\;\;\;\;\;\;\;\;\;\;\;\;\;\;\;\;\;\;\;\;\;\;\;\;\;\;\;\;\;\;\;\;\;\;\;\;\;\;\;\;\;\;\;\left(6\right)$$

where KL(U, X) is the Kullback–Leibler distance according to Eq. 5 and N is the total amount of bins.

#### GLM-CFC by Kramer and Eden (2013)

For this measure, the idea of predicting a set of observed variables (response variables; here the amplitude values of the relatively higher frequency band) by another set of variables (predictor variables; here the phase values of the relatively lower frequency band) using a mathematic function (link function; here a log link function) is applied. Extending the linear regression model, GLMs allow non-normal distributions for response variables (e.g., gamma distribution) and non-linear link functions (e.g., log link). They are thereby optimal for phase-amplitude coupling: phase and amplitude do exhibit a non-linear relationship and instantaneous amplitude values (extracted from the amplitude envelope) are always real and positive, which is best reflected in the gamma distribution (but not the normal distribution).

For calculating the GLM-CFC, phase is extracted from the low frequency filtered analytic signal and amplitude is extracted from the high frequency filtered analytic signal. Phase and amplitude values can then be depicted in a scatter plot (compare Figure 1B, outer right panels). If there is phase-amplitude coupling in the data, then amplitude values are specifically high at certain phase values. If there is no phase-amplitude coupling, amplitude values are rather similar across all possible phase values. In this case, a horizontal line would best model the data and the phase value would have no predictive power. If there is phase-amplitude coupling in the data, a curve (3^rd^ order polynomial) that follows the amplitude pattern would best model the data.

In case of phase-amplitude coupling, the curve – which is called spline model – (red lines in Figure 1B, outer right panels) differs from the horizontal line that represents no coupling – and is called null model – (black lines in Figure 1B, outer right panels). In case of no phase-amplitude coupling the spline model barely differs from the null model. That is, the more the spline model differs from the null model, the more phase-amplitude coupling is present in the data. In fact, the GLM-CFC finds the maximum absolute difference between both models, and calculates this difference as percentage change.

The modeled curve closely resembles a 3^rd^ order polynomial. However, not a polynomial, but a set of splines placed between control points, which are evenly spaced between –pi and pi, are used. The set of splines are easier to compute and, moreover, its characteristics can be better controlled than those of a polynomial. On the other hand, a degree of freedom is introduced (the amount of control points), that can influence the results. Kramer and Eden (2013) therefore included an evaluation of the Akaike information criterion (AIC) to define the optimal number of control points.

For an exact mathematical description of the GLM-CFC see the original article by Kramer and Eden (2013), who also provide the MATLAB code that was used in this analysis.

#### Permutation Testing

All methods are subjected to permutation testing in order to quantify the meaningfulness of the derived value (Cohen, 2014). For permutation testing, the observed coupling value is compared to a distribution of shuffled coupling values. Shuffled coupling values are constructed by calculating the coupling value between the original phase time series and a permuted amplitude time series (or vice versa). The permuted amplitude time series is constructed by cutting the amplitude time series at a random data point and reversing the order of both parts. Generating surrogate data this way is most conservative, because it leaves all characteristics of the EEG data intact, except the studied one, namely the temporal relationship between phase angle and amplitude magnitude. Shuffling is usually repeated 200 to 1000 times (here we used 1000). The observed coupling value is standardized to the distribution of the shuffled coupling values according to the following formula:

$${\text{CV}}_{\text{z}}\text{ = }\frac{{\text{CV}}_{\text{observed}}–{\text{μ}}_{{\text{CV}}_{\text{shuffled}}}}{{\text{σ}}_{{\text{CV}}_{\text{shuffled}}}}\;\;\;\;\;\;\;\;\;\;\;\;\;\;\;\;\;\;\;\left(7\right)$$

where CV denotes coupling value, μ denotes the mean and σ denotes the standard deviation (SD). Only when the observed CV is larger than 95% of shuffled values (which are expected to be uncorrelated), it is defined as significant.

### Statistical Analyses

All statistical analyses were conducted with IBM Statistics for Windows Version 23 (SPSS, Inc., IBM company), except otherwise specified. Significance level were set to *p* < 0.05. Violations of sphericity were, whenever appropriate corrected by Greenhouse–Geisser 𝜀 (Geisser and Greenhouse, 1958). Further analyses of significant results were conducted *post hoc* with Dunn’s multiple comparison procedure (Dunn, 1961) or *post hoc* *t*-tests. Effect size measure ω^2^ is reported for significant results (Hays, 1973). It is an estimator for the population effect Ω^2^, which specifies the systematic portion of variance in relation to the overall variance (Rasch et al., 2006).

#### Specificity of Phase-Amplitude Coupling Measures

In a first step 5,000 data sets without coupling were simulated by setting the modulation strength to I = 0. Simulations were carried out for the frequency pairs 5–7 Hz/33–47 Hz (for phase and amplitude time series respectively) and 8–10 Hz/50–70 Hz (for phase and amplitude time series respectively). Each data set was modified in data length (400, 2500, 5000 ms), sampling rate (500, 1000 Hz), and noise level (90, 100, 110%), resulting in a total of 90,000 data sets for which coupling was calculated. Phase-amplitude coupling values were generally compared in a 4 × 3 × 2 × 3 analysis of variance (ANOVA) with the repeated measurement factors method (PLV, MVL, MI, GLM-CFC), data length (400, 2500, 5000 ms), sampling rate (500, 1000 Hz), and noise level (90, 100, 110%).

As described above, non-parametric permutation testing was performed. Raw phase-amplitude coupling measures were z-standardized to the shuffled phase-amplitude coupling distribution. Normal z-values directly imply *p*-values; a value of 1.64 corresponds to a *p*-value of 5%. The phase-amplitude coupling value distribution which is expected under the null-hypothesis does not have to match the standardized normal distribution. Therefore, significance was not inferred from the standardized normal distribution, but instead by that phase-amplitude coupling value, at which 5% of simulated data (with no coupling) was classified as false positive. Shuffling for permutation testing was done within trials. Coupling measures were then calculated on concatenated trials.

Specificity of measures was analyzed by counting false positives (significant coupling according to critical z-value found in the prior analysis, even though it was not engineered into the simulated data) depending on (1) method, (2) data length, (3) sampling rate, and (4) noise level. To be able to conduct an ANOVA, the 5,000 simulations were divided into 100 subsamples of 50 simulations each. For each subsample false positives were counted. Each subsample was treated as a case in the subsequent 4 × 2 × 3 × 3 ANOVA with the repeated measurement factors method (PLV, MVL, MI, GLM-CFC), data length (400, 2500, 5000 ms), sampling rate (500, 1000 Hz), and noise level (90, 100, 110%) and the dependent variable false positives.

#### Sensitivity of Phase-Amplitude Coupling Measures as a Function of Moderating Variables

Performance of phase-amplitude coupling measures were quantified by simulating 100 independent data sets and modifying the parameters (1) modulation strength, and (2) modulation width, (3) multimodality, (4) data length, (5) sampling rate, and (6) noise level within each dataset. Six two-way ANOVAs were calculated. Each ANOVA included the repeated measurement factor method and was individually combined with the repeated measurement factors modulation strength (90, 100, 110%), modulation width (22.5, 25.0, 27.5% of one low frequency cycle), multimodality (monophasic, biphasic), data length (400, 2500, 5000 ms), sampling rate (500, 1000 Hz), and noise level (90, 100, 110% compared to signal strength).

## Results

### Specificity of Phase-Amplitude Coupling Measures

#### Theta-Low Gamma Coupling (5–7 to 33–47 Hz)

Phase-amplitude coupling values did not differ depending on data length, sampling rate, or noise level. Because of the high number of simulations (*n* = 5,000), some other main effects and interactions became significant. However, all effect sizes were below ω^2^ < 0.01, therefore these differences are negligible. Phase-amplitude coupling values did differ depending on method [*F*(3,14997) = 4471.38, *p* < 0.01, ω^2^ = 0.40]. *Post hoc* *t*-tests showed that the GLM-CFC (mean ± SE: 0.29 ± 0.00) was significantly larger than all other methods [PLV:.02 ± 0.00, *t*(4999) = 74.75, *p* < 0.01, ω^2^ = 0.36; MVL: 0.02 ± 0.00, *t*(4999) = 78.09, *p* < 0.01, ω^2^ = 0.38; MI: 0.00 ± 0.00, *t*(4999) = 187.48, *p* < 0.01, ω^2^ = 0.78], which did not differ significantly from each other (all ω^2^ < 0.01).

Five percent of the simulated data were falsely classified as containing coupling when setting the critical z-value for the PLV at 1.91, for the MVL at 1.91, for the MI at 1.94, and for the GLM-CFC at 2.08. Thus, these values were defined as critical z-values. This implies that the PLV and the MVL are most specific, followed by the MI. The GLM-CFC is least specific compared to the three other methods.

The amount of false positives according to the previous established critical z-value did differ depending on data length [*F*(2,198) = 35.57, *p* < 0.01, ω^2^ = 0.19, Dunn_crit_ = 0.14]. There were significantly more false positives during short epochs (400 ms; 2.77 ± 0.04) compared to medium (2500 ms: 2.36 ± 0.04) and long epochs (5000 ms: 2.32 ± 0.05). Medium and long epochs did not differ in their false positive rates. The main effect was qualified by a method by data length interaction [*F*(6,594) = 51.66, *p* < 0.01, ω^2^ = 0.20, Dunn_crit_ = 0.20]. This revealed that the above-described pattern was driven by the PLV and MVL. There were no differences in false positive rate within the MI and the GLM-CFC.

#### Alpha-High Gamma Coupling (8–10 to 50–70 Hz)

Phase-amplitude coupling values did not differ depending on data length, sampling rate, or noise level. Because of the high number of simulations (*n* = 5,000), some other main effects and interactions became significant. However, all effect sizes were below ω^2^ < 0.01, therefore these differences are negligible. Phase-amplitude coupling values did differ depending on method [*F*(3,14997) = 3959.41, *p* < 0.01, ω^2^ = 0.37]. *Post hoc* *t*-tests showed that the GLM-CFC (0.24 ± 0.00) was significantly larger than all other methods [PLV: 0.01 ± 0.00, *t*(4999) = 70.29, *p* < 0.01, ω^2^ = 0.33; MVL: 0.01 ± 0.00, *t*(4999) = 75.56, *p* < 0.01, ω^2^ = 0.36; MI: 0.00 ± 0.00, *t*(4999) = 161.05, *p* < 0.01, ω^2^ = 0.72], which did not differ significantly from each other (all ω^2^ < 0.01).

**FIGURE 2:**
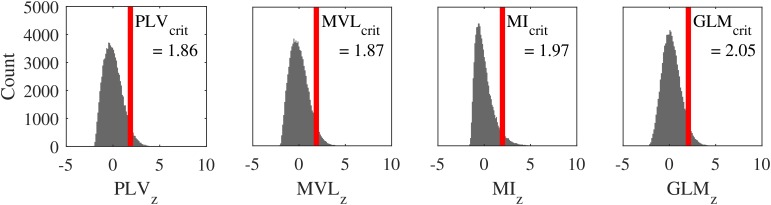
Probability distribution of coupling values under the null hypothesis: phase-amplitude coupling value distribution under the null hypothesis (i.e., no coupling present in the data) of phase-locking value (outer left panel), MVL (inner left panel), MI (inner right panel), and GLM-CFC (outer right panel). These distributions allow defining the significance threshold. The red line marks the critical phase-amplitude coupling z-value (relative cut off of 5%). Choosing an absolute cut off instead would lead to smallest amount of false positives for MVL, followed by the PLV. The GLM-CFC would detect the most false positives followed by the MI.

Figure 2 shows the phase-amplitude coupling value distribution for the PLV, the MVL, the MI, and the GLM-CFC for alpha-high gamma coupling. Five percent of the simulated data were falsely classified as containing coupling when setting the critical z-value for the PLV at 1.86, for the MVL at 1.87, for the MI at 1.97, and for the GLM-CFC at 2.05. Thus, these values were defined as critical z-values. This implies that the PLV and the MVL are most specific, followed by the MI. The GLM-CFC is least specific compared to the three other methods.

The amount of false positives according to the previous established critical z-value did differ depending on data length [*F*(2,198) = 4.72, *p* < 0.01, ω^2^ = 0.02, Dunn_crit_ = 0.17]. There were significantly more false positives during short epochs (400 ms: 2.62 ± 0.05) compared to medium (2500 ms: 2.42 ± 0.05) and long epochs (5000 ms: 2.43 ± 0.05). Medium and long epochs did not differ in their false positive rates. The main effect was qualified by a method by data length interaction [*F*(6,594) = 13.28, *p* < 0.01, ω^2^ = 0.06, Dunn_crit_ = 0.18]. This revealed that the above-described pattern was driven by the PLV and MVL. There were no differences in false positive rate within the MI and the GLM-CFC.

### Sensitivity of Phase-Amplitude Coupling Measures as a Function of Moderating Variables

#### Effect of Method on Phase-Amplitude Coupling Measures

##### Theta-low gamma coupling (5–7 to 33–47 Hz)

PLV (1.22 ± 0.05) and MVL (1.53 ± 0.06) differed from the MI (7.83 ± 0.49) in their absolute magnitude independently of any other factor [main effect method: *F*(3,297) = 220.33, *p* < 0.01, ω^2^ = 0.62, Dunn_crit_ = 0.78]. PLV and MVL did not differ from each other. The GLM-CFC (3.91 ± 0.18) differed from all other methods.

##### Alpha-high gamma coupling (8–10 to 50–70 Hz)

PLV (1.77 ± 0.06) and MVL (2.22 ± 0.08) differed from the MI (13.35 ± 0.78) in their absolute magnitude independently of any other factor [main effect method: *F*(3,297) = 250.07, *p* < 0.01, ω^2^ = 0.65, Dunn_crit_ = 1.28]. PLV and MVL did not differ from each other. The GLM-CFC (5.52 ± 0.23) differed from all other methods.

#### Effect of Modulation Strength on Phase-Amplitude Coupling Measures

##### Theta-low gamma coupling (5–7 to 33–47 Hz)

Coupling values of all methods increased with increasing modulation strength [*F*(2,198) = 204.74, *p* < 0.01, ω^2^ = 0.58]. The interaction method by modulation strength became significant [*F*(6,594) = 154.84, *p* < 0.01, ω^2^ = 0.43]. *Post hoc* *t*-tests showed that all factor levels within a method differed significantly from each other (all *p*’s < 0.01). The effect of modulation strength was most pronounced for the GLM-CFC (0.31 < ω^2^ < 0.61), followed by the MVL (0.21 < ω^2^ < 0.55) and the MI (0.33 < ω^2^ < 0.54). The PLV was least sensitive to modulation strength (0.15 < ω^2^ < 0.50).

##### Alpha-high gamma coupling (8–10 to 50–70 Hz)

**FIGURE 3:**
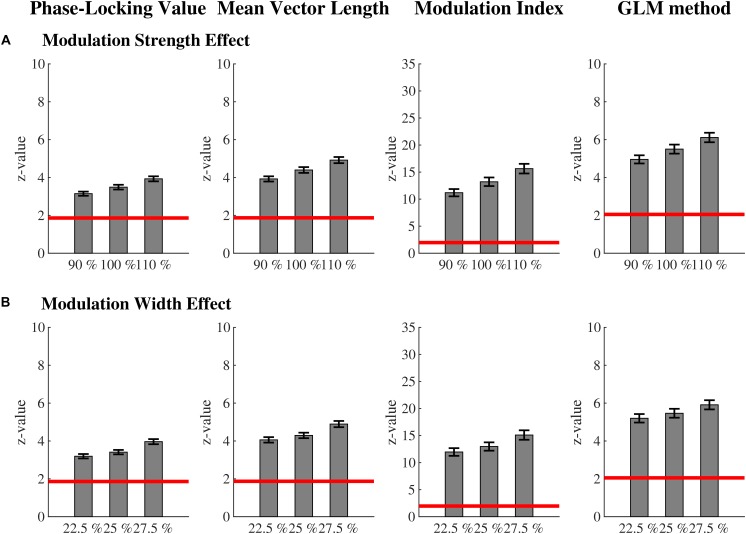
Sensitivity for modulation strength and width: mean ( ± SEM) phase-amplitude coupling values for each method for the **(A)** modulation strength effect and **(B)** modulation width effect. Coupling values of all methods increased with increasing modulation strength. However, in addition to the GLM-CFC, MVL differentiates best between the different factor levels of modulation strength. Also, coupling values of all methods increased with increasing modulation width. Here, PLV and MVL differentiate best between the different factor levels of modulation width. The red line marks the critical z-value (significance level). All values above this line represent significant phase-amplitude coupling. For each effect, all factor levels within a method are significantly different from each other according to *post hoc t*-tests. Only monophasic coupling values are depicted for the PLV and the MVL.

Coupling values of all methods increased with increasing modulation strength [*F*(2,198) = 215.60, *p* < 0.01, ω^2^ = 0.59]. The interaction method by modulation strength became significant [*F*(6,594) = 167.31, *p* < 0.01, ω^2^ = 0.45; Figure 3A]. *Post hoc t*-tests showed that all factor levels within a method differed significantly from each other (all *p*’s < 0.01). The effect of modulation strength was most pronounced for the GLM-CFC (0.36 < ω^2^ < 0.66) and the MVL (0.32 < ω^2^ < 0.66), followed by the MI (0.33 < ω^2^ < 0.57). The PLV was least sensitive to modulation strength (0.20 < ω^2^ < 0.60).

The stronger the coupling, the larger PLV, MVL, MI, and GLM-CFC are. As Tort et al. (2010) has shown, this behavior is not inherent to all phase-amplitude coupling measures. Since researchers do not only want to prove the existence of phase-amplitude coupling, but also differentiate its strength, a measure that can do this is indispensable. Of all four methods, the GLM-CFC differentiates best between the different factor levels of modulation strength, closely followed by the MVL and MI.

#### Effect of Modulation Width on Phase-Amplitude Coupling Measures

##### Theta-low gamma coupling (5–7 to 33–47 Hz)

Coupling values of all methods increased with increasing modulation width [*F*(2,198) = 118.61, *p* < 0.01, ω^2^ = 0.44]. The interaction method by modulation width became significant [*F*(6,594) = 79.45, *p* < 0.01, ω^2^ = 0.28]. *Post hoc t*-tests showed that all factor levels within a method differed significantly from each other (all *p*’s < 0.01). The effect of modulation width was most pronounced for the MVL (0.19 < ω^2^ < 0.51), followed by the PLV (0.22 < ω^2^ < 0.48). MI (0.15 < ω^2^ < 0.44) and GLM-CFC (0.11 < ω^2^ < 0.45) were least sensitive to modulation width.

##### Alpha-high gamma coupling (8–10 to 50–70 Hz)

Coupling values of all methods increased with increasing modulation width [*F*(2,198) = 145.07, *p* < 0.01, ω^2^ = 0.49]. The interaction method by modulation width became significant [*F*(6,594) = 103.84, *p* < 0.01, ω^2^ = 0.34; Figure 3B]. *Post hoc t*-tests showed that all factor levels within a method differed significantly from each other (all *p*’s < 0.01). The effect of modulation width was most pronounced for the MVL (0.11 < ω^2^ < 0.57), followed by the PLV (0.10 < ω^2^ < 0.54) and the GLM-CFC (0.11 < ω^2^ < 0.53). The MI was least sensitive to modulation width (0.12 < ω^2^ < 0.47).

The broader the coupling width, the larger PLV, MVL, MI, and GLM-CFC are. Of all four methods, MVL differentiates best between the different factor levels of modulation width.

#### Effect of Multimodality on Phase-Amplitude Coupling Measures

##### Theta-low gamma coupling (5–7 to 33–47 Hz)

Monophasic coupling (4.89 ± 0.24) led to overall stronger coupling measures than biphasic coupling [2.36 ± 0.15; *F*(1,99) = 586.81, *p* < 0.01, ω^2^ = 0.75]. This interaction was further qualified by method [*F*(3,297) = 73.81, *p* < 0.01, ω^2^ = 0.21]. Biphasic coupling could not be detected by the PLV [2.42 ± 0.10 vs. 0.02 ± 0.01; *t*(99) = 25.20, *p* < 0.01, ω^2^ = 0.76] and MVL [3.04 ± 0.12 vs. 0.02 ± 0.01; *t*(99) = 25.54, *p* < 0.01, ω^2^ = 0.77]. The MI was larger in monophasic than in biphasic coupling [9.24 ± 0.53 vs. 6.41 ± 0.45; *t*(99) = 18.54, *p* < 0.01, ω^2^ = 0.63]. The GLM-CFC was as well larger in monophasic than in biphasic coupling [4.86 ± 0.22 vs. 2.96 ± 0.14; *t*(99) = 21.90, *p* < 0.01, ω^2^ = 0.71].

##### Alpha-high gamma coupling (8–10 to 50–70 Hz)

**FIGURE 4:**
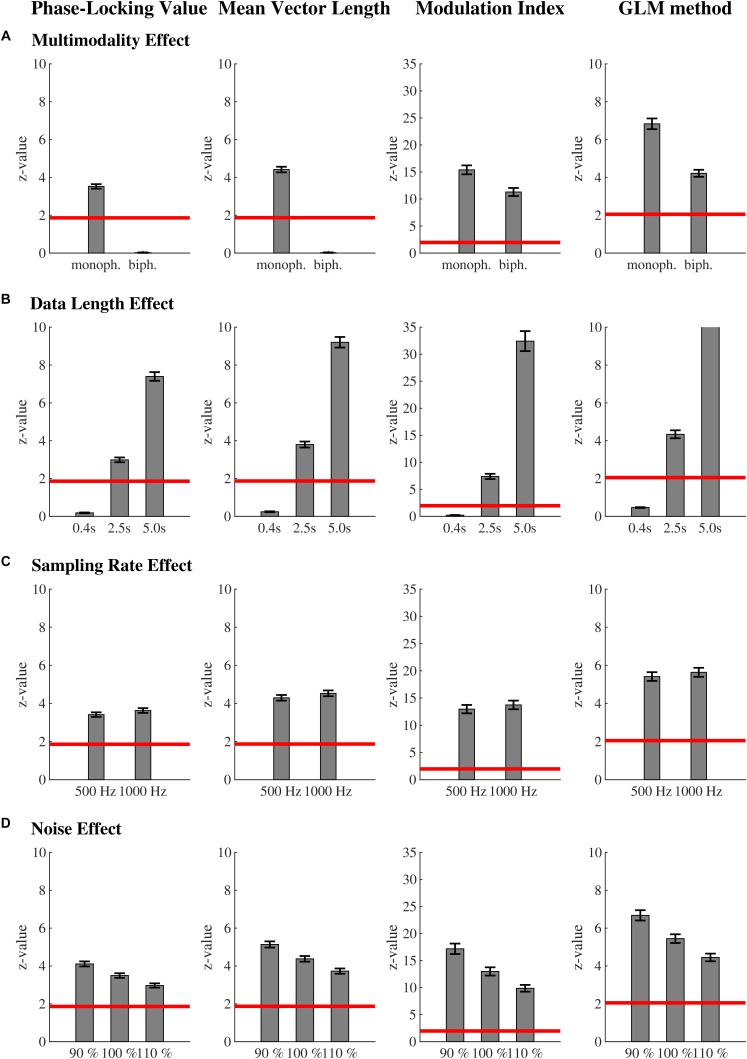
Moderators of the phase-amplitude coupling measures: mean ( ± SEM) phase-amplitude coupling values for each method for the **(A)** multimodality effect, **(B)** data length effect, **(C)** sampling rate effect, and **(D)** noise effect. In contrast to the MI and GLM-CFC, biphasic coupling could not be detected by the PLV and MVL. This factor might turn out to be not as important, as most studies report monophasic coupling. Coupling values of all methods increased with increasing data length and slightly increase with sampling rate. Sampling rate only becomes relevant when analyzing frequencies close to the Nyquist frequency. Of all four methods, MI is least affected from the confounding factor data length. Coupling values of all methods decreased with increasing noise, while the PLV is least affected from this confounding factor. The red line marks the critical z-value (significance level). All values above this line represent significant phase-amplitude coupling. For each effect, all factor levels within a method are significantly different from each other according to *post hoc t*-tests. For **(B–D)** only monophasic coupling values are depicted for the PLV and the MVL.

Monophasic coupling (7.54 ± 0.34) led to overall stronger coupling measures than biphasic coupling [3.89 ± 0.23; *F*(1,99) = 782.07, *p* < 0.01, ω^2^ = 0.80]. This interaction was further qualified by method [*F*(3,297) = 74.41, *p* < 0.01, ω^2^ = 0.22]. Biphasic coupling could not be detected by the PLV [3.52 ± 0.12 vs. 0.02 ± 0.01; *t*(99) = 29.27, *p* < 0.01, ω^2^ = 0.81] and MVL [4.41 ± 0.15 vs. 0.02 ± 0.01; *t*(99) = 29.57, *p* < 0.01, ω^2^ = 0.81]. The MI was larger in monophasic than in biphasic coupling [15.40 ± 0.83 vs. 11.29 ± 0.74; *t*(99) = 19.22, *p* < 0.01, ω^2^ = 0.65]. The GLM-CFC was as well larger in monophasic than in biphasic coupling [6.83 ± 0.28 vs. 4.22 ± 0.19; *t*(99) = 24.78, *p* < 0.01, ω^2^ = 0.75; Figure 4A].

That is, multimodality influences the four methods very differently. PLV and MVL cannot find biphasic coupling as it was implemented here (amplitude of the higher frequency was increased at peak and trough of the lower frequency). Because of the mathematic construct of the MVL (Eq. 2, Figure 1B) this is not surprising. Peak and trough appear on opposite sides in the polar plane: their mean will cancel each other out. If other forms of biphasic coupling would be present, the MVL could be able to find it, but would probably underestimate its strength and would furthermore return distorted phase information. Therefore, it is important to have a look at the polar plot before interpreting one’s results. Similarly, the PLV cannot detect biphasic coupling, as it was implemented here. For biphasic coupling the amplitude envelope oscillates twice as fast as the lower frequency band. Because of this, the phase lag between lower and upper frequency band spans the entire polar plane. The MI and GLM-CFC are able to find biphasic coupling, but biphasic coupling leads to a reduction in the phase-amplitude coupling value; this undesirable reduction is stronger for the GLM-CFC than for the MI. Literature indicates that biphasic coupling plays a minor role in empirical data. To our knowledge only a very small fraction of studies report biphasic coupling (e.g., van der Meij et al., 2012; Leszczynski et al., 2015; Lega et al., 2016). Most studies report monophasic coupling (e.g., Tort et al., 2008).

#### Effect of Data Length on Phase-Amplitude Coupling Measures

##### Theta-low gamma coupling (5–7 to 33–47 Hz)

Coupling values of all methods increased with increasing data length [main effect data length: *F*(2,198) = 390.95, *p* < 0.01, ω^2^ = 0.72]. For the shortest epoch of 400 ms, none of the methods could detect significant coupling, even though it was engineered into the data. The interaction method by data length [*F*(6,594) = 251.91, *p* < 0.01, ω^2^ = 0.56] became significant. *Post hoc t*-tests showed that all factor levels within a method differed significantly from each other (all *p*’s < 0.01). The data length effect was most pronounced for MVL (0.60 < ω^2^ < 0.85), and PLV (0.57 < ω^2^ < 0.83), followed by the GLM-CFC (0.56 < ω^2^ < 0.77). The MI was least affected by data length (0.46 < ω^2^ < 0.62).

##### Alpha-high gamma coupling (8–10 to 50–70 Hz)

Coupling values of all methods increased with increasing data length [main effect data length: *F*(2,198) = 422.16, *p* < 0.01, ω^2^ = 0.74]. For the shortest epoch of 400 ms, none of the methods could detect significant coupling, even though it was engineered into the data. The interaction method by data length [*F*(6,594) = 270.73, *p* < 0.01, ω^2^ = 0.57; Figure 4B] became significant. *Post hoc t*-tests showed that all factor levels within a method differed significantly from each other (all *p*’s < 0.01). The data length effect was most pronounced for MVL (0.75 < ω^2^ < 0.87), and PLV (0.73 < ω^2^ < 0.86), followed by the GLM-CFC (0.66 < ω^2^ < 0.79). The MI was least affected by data length (0.54 < ω^2^ < 0.62).

Overall, the longer the data, the larger PLV, MVL, MI, and GLM-CFC are. This association was found in the data presented here, but must not generally apply. Here coupling was simulated continuously into the data. If coupling is transient and does not proportionally vary with data length, this relationship does not need to apply. Penny et al. (2008) showed, that coupling strength decreases for phase-amplitude coupling, which was simulated transiently. Potentially, the general rule is that the longer the data epochs where coupling occurs, the stronger the phase-amplitude coupling values. This should be tested in a follow-up analysis. This analysis further showed that a minimal data length is required for finding coupling, which should exceed at least 400 ms per trial when including 30 trials (also see Cheng et al., 2018). None of the methods were able to detect coupling in the shortest simulated epoch of 400 ms. It might be useful to develop a correction factor (e.g., similar to the pairwise phase consistency that is insensitive to data length variation; Vinck et al., 2010) for data length, to make phase-amplitude coupling values more comparable across studies. Of all four methods, MI is least affected from the confounding factor data length.

#### Effect of Sampling Rate on Phase-Amplitude Coupling Measures

##### Theta-low gamma coupling (5–7 to 33–47 Hz)

Sampling rate had no effect on any of the phase-amplitude coupling values [*F*(1,99) = 0.10, *p* = 0.75] and did not interact with method [*F*(3,297) = 2.05, *p* = 0.15].

##### Alpha-high gamma coupling (8–10 to 50–70 Hz)

Overall coupling values slightly increased with increasing sampling rate [*F*(1,99) = 38.65, *p* < 0.01, ω^2^ = 0.16]. The sampling rate effect differed according to the method [*F*(3,297) = 27.80, *p* < 0.01, ω^2^ = 0.09; Figure 4C]. It was most pronounced in GLM-CFC [*t*(99) = 6.26, *p* < 0.01, ω^2^ = 0.16], followed by the MI [*t*(99) = 5.71, *p* < 0.01, ω^2^ = 0.14]. PLV [*t*(99) = 5.31, *p* < 0.01, ω^2^ = 0.12] and mean vector [*t*(99) = 5.28, *p* < 0.01, ω^2^ = 0.12] length were least affected by sampling rate.

The factor sampling rate stands out because of its lacking effect for theta-low gamma coupling and comparatively small effect size for alpha-high gamma coupling. A third set of data was simulated testing PLV, MVL, and MI at 16–18 Hz for the modulating frequency and 202–238 Hz for the modulated frequency (for detailed results see Hülsemann, 2016). This analysis showed that sampling rate is indeed important, but only if the investigated upper frequency band approaches the Nyquist frequency (here 250 Hz). Of all four methods, MVL and PLV are least affected from the confounding factor sampling rate.

#### Effect of Noise on Phase-Amplitude Coupling Measures

##### Theta-low gamma coupling (5–7 to 33–47 Hz)

Coupling values of all methods decreased with increasing noise [*F*(2,198) = 372.07, *p* < 0.01, ω^2^ = 0.71]. The interaction method by noise became significant [*F*(6,594) = 247.63, *p* < 0.01, ω^2^ = 0.55]. *Post hoc t*-tests showed that all factor levels within a method differed significantly from each other (all *p*’s < 0.01). The effect of noise was most pronounced for the GLM-CFC (0.65 < ω^2^ < 0.75). MVL (0.42 < ω^2^ < 0.70) and MI (0.53 < ω^2^ < 0.62) were intermediately affected. The PLV (0.30 < ω^2^ < 0.65) was least affected by noise.

##### Alpha-high gamma coupling (8–10 to 50–70 Hz)

Coupling values of all methods decreased with increasing noise [*F*(2,198) = 417.74, *p* < 0.01, ω^2^ = 0.74]. The interaction method by noise became significant [*F*(6,594) = 290.04, *p* < 0.01, ω^2^ = 0.59; Figure 4D]. *Post hoc t*-tests showed that all factor levels within a method differed significantly from each other (all *p*’s < 0.01). The effect of noise was most pronounced for the GLM-CFC (0.67 < ω^2^ < 0.79). MVL (0.50 < ω^2^ < 0.80) and MI (0.55 < ω^2^ < 0.66) were intermediately affected. The PLV (0.44 < ω^2^ < 0.76) was least affected by noise.

Overall, the noisier the data, the lower PLV, MVL, MI, and GLM-CFC are. This aspect is not desired but plausible. Noise obscures the relation between the phase of the lower frequency and amplitude of the higher frequency. The data as a whole contains phase-amplitude coupling to a lesser extent, as the relative amount of noise compared to the relative amount of signal increases. Of all four methods, the GLM-CFC is most and the PLV is least affected from the confounding factor noise. The MVL is stronger affected than the MI.

#### Interaction Effects

**FIGURE 5:**
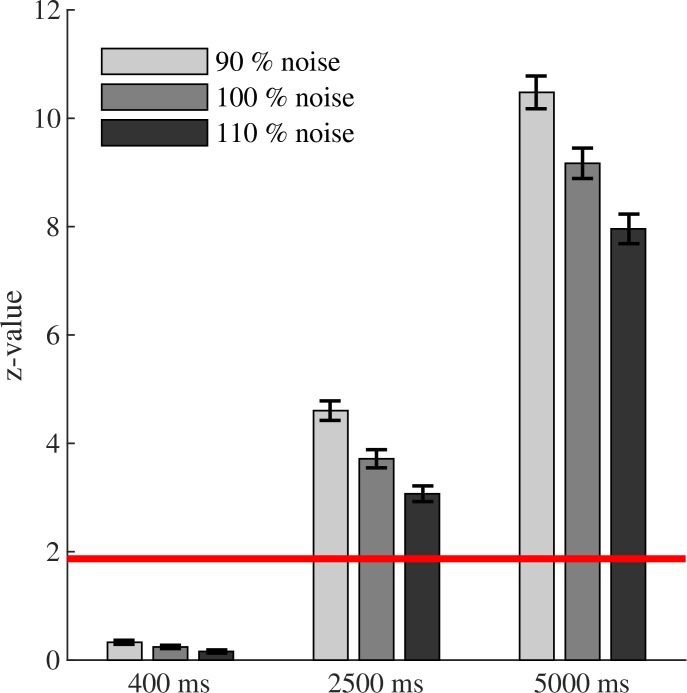
Interaction effects between the moderators of the phase-amplitude coupling measures: mean ( ± SEM) phase-amplitude coupling values for the MVL for the data length by noise interaction (only monophasic coupling values). Interactions had a monotone pattern, following the pattern of each main effect. Depicted here, MVL increased the longer the data, but it increased less when also noise increased. This pattern was true for each added factor. The red line marks the critical z-value (significance level). All values above this line represent significant phase-amplitude coupling. For each method, all factor levels are significantly different from each other according to Dunn’s *post hoc* test. Only values within the 400 ms condition do not differ between the noise levels.

Conducting six-way ANOVAs for each method separately (see Hülsemann, 2016 for detailed results), revealed ordinal interaction for all factors (multimodality, data length, sampling rate, noise, modulation strength, and modulation width). Especially multimodality and data length interacted with the remaining factors, as well as interacted with each other and the remaining factors. Sampling rate only showed significant interactions when analyzing frequencies close to the Nyquist frequency. All interactions had a monotone pattern, following the pattern of each main effect. For example, MVL increased the longer the data, but it increased less when also noise increases (Figure 5). This pattern was true for each added factor. Phase-locking value and MVL did not find biphasic coupling at all. Because of this, for these two methods, the described main effect and interaction patterns are only valid for monophasic, but not for biphasic coupling. For the MI and the GLM-CFC the pattern was true for mono- and for biphasic coupling.

We showed empirically that the methods were indifferent to the chosen frequency band combinations. To our knowledge, there is no mathematical reason for a frequency dependency of the methods [compare Figure 1 showing calculation of all methods graphically and see sections “Phase-Locking-Value as Used in Mormann et al. (2005),” Mean Vector Length by Canolty et al. (2006),” Modulation Index by Tort et al. (2008),” and “GLM-CFC by Kramer and Eden (2013)”]. In order to facilitate the testing of methods, we provide our MATLAB script in Appendix A, in which the chosen frequency band combination and parameters can easily be adjusted.

The GLM-CFC behaves best regarding modulation strength and worst regarding noise compared with the three other methods. Regarding the other factors, its performance is in the intermediate range. The most important disadvantage of the GLM-CFC is its extremely high computation time, which exceeds those of the other methods by two (without calculating confidence intervals) or up to four orders of magnitude (with calculating confidence intervals). On a Windows 10 computer (64-bit operating system, CPU: Intel^®^ Core ^TM^i7-8700K, CPU ^2^ 3.70 GHz 3.70 GHz, RAM: 16.0 GB) the calculation took on average 0.61 ms for the PLV, 0.67 ms for the MVL, 2.70 ms for the MI, 269.51 ms for the GLM-CFC (excluding the built-in confidence interval calculation), and 9159.08 ms for the GLM-CFC (including the built-in confidence interval calculation). Increasing data points increases computation time for all methods in a similar manner (e.g., doubling the data points doubles the computation time). Assuming, that this time-factor will lead to the exclusion of this method for most researchers, it is not further considered in the conclusion of this manuscript. For a more detailed review of this method, see Kramer and Eden (2013).

Comparing the remaining three methods it becomes evident that the MI is least affected by the confounding factors multimodality and data length. However, it is also – like the PLV – less sensitive to variation in modulations strength compared with the MVL. The MI is especially less sensitive to modulation width compared to the MVL and PLV. MVL and MI are similarly – and stronger than the PLV – affected by the confounding factor noise.

## Conclusion

For long data epochs, recorded at high sampling rates, with a high signal-to-noise ratio, the use of the MVL is recommended, because it is more sensitive to modulation strength and width than both other methods. For noisier data, shorter data epochs, recorded at a lower sampling rate, the use of the MI is recommended, as it is least influenced by the confounding factors compared with both other methods. If it is not clear whether cross-frequency coupling will be mono- or bi-phasic, the MI should be used, even though literature suggests that biphasic coupling can be neglected.

The PLV does not stand out in comparison to the two other measures. So far, no review evaluated this measure explicitly as positive. Its usage is potentially problematic because phase information is extracted from the amplitude envelope of a signal. Phase information can only be correctly extracted from truly oscillating signals; this must not be necessarily the case for an amplitude envelope. However this disadvantage can be counteracted by filtering the amplitude envelope first before extracting phase information from it as is described (Vanhatalo et al., 2004).

Because MVL and MI have complementing strengths and weaknesses, it would be advisably to calculate both. The time-consuming aspect of measuring the two methods is permutation testing. Calculation of both measures on the other hand will not substantially increase the analysis time.

The MI is quantitatively larger than the PLV and MVL. However, even despite substantial quantitative differences in values, the qualitative decision for significance of phase-amplitude coupling is the same for all four methods in our simulation. Nevertheless, comparison of coupling strengths between the methods is problematic and this lack of comparability provides another reason for reporting both, MVL and MI.

In contrast to MVL, the false positive rate of the MI is not affected by any confounding factor. However, this advantage against MVL is counteracted by one disadvantage against the MVL: calculation of the MI includes Shannon’s Entropy. The entropy value depends on the amount of bins as well as amount of data squeezed into the same amount of bins. This is an undesirable degree of freedom, which is not present when calculating the MVL.

Due to the dependency on confounding variables (e.g., data length), comparing absolute coupling strengths across studies might be difficult even if using the same method. Comparisons within one study, on the other hand, can be done with confidence. Nevertheless, one should make sure that signal-to-noise ratio is comparable within all experimental conditions and over the course of the experiment.

Generally, it is advisable to work with standardized phase-amplitude coupling measures via permutation testing. It facilitates the interpretation of the measures, first and foremost, by giving the researcher knowledge about the probability that the observed MI would have been also found under the assumption of the null-hypothesis. This aspect is often ignored in the literature.

Kramer and Eden (2013) stated that “an optimal analysis method to assess this cross-frequency coupling (CFC) does not yet exist” (p. 64). Even if it would be ideal, to have a measure that is less susceptible to confounding variables summarizing this analysis, it should be rather concluded that at least two reasonable analysis methods exist.

## Author Contributions

MH conducted the data simulation and performed the statistical analysis. MH, EN, and BR wrote the manuscript. All authors contributed to manuscript revision, read and approved the submitted version.

## Conflict of Interest Statement

The authors declare that the research was conducted in the absence of any commercial or financial relationships that could be construed as a potential conflict of interest.

## References

[B1] Aru J. Aru J. Priesemann V. Wibral M. Lana L. Pipa G. (2015). Untangling cross-frequency coupling in neuroscience. *Cur. Opin. Neurobiol.* 31 51–61. 10.1016/j.conb.2014.08.002 25212583

[B2] Berman J. I. McDaniel J. Liu S. Cornew L. Gaetz W. Roberts T. P. (2012). Variable bandwidth filtering for improved sensitivity of cross-frequency coupling metrics. *Brain Connect.* 2 155–163. 10.1089/brain.2012.0085 22577870 PMC3621836

[B3] Bruns A. Eckhorn R. (2004). Task-related coupling from high- to low-frequency signals among visual cortical areas in human subdural recordings. *Int. J. Psychophysiol.* 51 97–116. 10.1016/j.ijpsycho.2003.07.001 14693360

[B4] Burgess A. P. Ali L. (2002). Functional connectivity of gamma EEG activity is modulated at low frequency during conscious recollection. *Int. J. Psychophysiol.* 46 91–100. 10.1016/S0167-8760(02)00108-3 12433386

[B5] Canolty R. T. Edwards E. Dalal S. S. Soltani M. Nagarajan S. S. Kirsch H. E. (2006). High gamma power is phase-locked to theta oscillations in human neocortex. *Science* 313 1626–1628. 10.1126/science.1128115 16973878 PMC2628289

[B6] Cheng N. Li Q. Wang S. Wang R. Zhang T. (2018). permutation mutual information: a novel approach for measuring neuronal phase-amplitude coupling. *Brain Topogr.* 31 186–201. 10.1007/s10548-017-0599-2 28983770

[B7] Cohen M. X. (2008). Assessing transient cross-frequency coupling in EEG data. *J. Neurosci. Methods* 168 494–499. 10.1016/j.jneumeth.2007.10.012 18061683

[B8] Cohen M. X. (2014). *Analyzing Neural Time Series Data: Theory and Practice.* Massachusetts, MA: The MIT Press.

[B9] Dunn O. J. (1961). Multiple comparisons among means. *J. Am. Statist. Assoc.* 56 52–64. 10.1080/01621459.1961.10482090

[B10] Dvorak D. Fenton A. A. (2014). Toward a proper estimation of phase-amplitude coupling in neural oscillations. *J. Neurosci. Methods* 225 42–56. 10.1016/j.jneumeth.2014.01.002 24447842 PMC3955271

[B11] Geisser S. Greenhouse S. W. (1958). An extension of box’s results on the use of the f distribution in multivariate analysis. *Ann. Math. Statist.* 29 885–891. 10.1214/aoms/1177706545

[B12] Hays W. L. (1973). *Statistics for the Social Sciences.* New York, NY: Holt, Rinehart and Winston.

[B13] He B. J. Zempel J. M. Snyder A. Z. Raichle M. E. (2010). The temporal structures and functional significance of scale-free brain activity. *Neuron* 66 353–369. 10.1016/j.neuron.2010.04.020 20471349 PMC2878725

[B14] Hülsemann M. J. (2016). *The Role of Phase-Amplitude Coupling in the Relationship between Acute Stress and Executive Functions.* [dissertation]. Trier: Universität Trier.

[B15] Jensen O. (2006). Maintenance of multiple working memory items by temporal segmentation. *Neuroscience* 139 237–249. 10.1016/j.neuroscience.2005.06.004 16337089

[B16] Jensen O. Lisman J. E. (1998). An oscillatory short-term memory buffer model can account for data on the Sternberg task. *J. Neurosci.* 18 10688–10699. 10.1523/jneurosci.18-24-10688.1998 9852604 PMC6793327

[B17] Kramer M. A. Eden U. T. (2013). Assessment of cross-frequency coupling with confidence using generalized linear models. *J. Neurosci. Methods* 220 64–74. 10.1016/j.jneumeth.2013.08.006 24012829 PMC3813466

[B18] Lakatos P. Shah A. S. Knuth K. H. Ulbert I. Karmos G. Schroeder C. E. (2005). An oscillatory hierarchy controlling neuronal excitability and stimulus processing in the auditory cortex. *J. Neurophysiol.* 94 1904–1911. 10.1152/jn.00263.2005 15901760

[B19] Lega B. Burke J. Jacobs J. Kahana M. J. (2016). Slow-theta-to-gamma phase-amplitude coupling in human hippocampus supports the formation of new episodic memories. *Cereb. Cortex* 26 268–278. 10.1093/cercor/bhu232 25316340 PMC4677977

[B20] Leszczynski M. Fell J. Axmacher N. (2015). Rhythmic working memory activation in the human hippocampus. *Cell Rep.* 13 1272–1282. 10.1016/j.celrep.2015.09.081 26527004

[B21] Lisman J. E. Jensen O. (2013). The theta-gamma neural code. *Neuron* 77 1002–1016. 10.1016/j.neuron.2013.03.007 23522038 PMC3648857

[B22] Luck S. J. (2014). *An Introduction to the Event-Related Potential Technique.* Cambridge: The MIT Press.

[B23] Maris E. van Vugt M. Kahana M. (2011). Spatially distributed patterns of oscillatory coupling between high-frequency amplitudes and low-frequency phases in human iEEG. *NeuroImage* 54 836–850. 10.1016/j.neuroimage.2010.09.029 20851192

[B24] Martínez-Cancino R. Heng J. Delorme A. Kreutz-Delgado K. Sotero R. C. Makeig S. (2019). Measuring transient phase-amplitude coupling using local mutual information. *NeuroImage* 185 361–378. 10.1016/j.neuroimage.2018.10.034 30342235 PMC6342492

[B25] Miller K. J. Sorensen L. B. Ojemann J. G. den Nijs M. (2009). Power-law scaling in the brain surface electric potential. *PLoS Computat. Biol.* 5:e1000609. 10.1371/journal.pcbi.1000609 20019800 PMC2787015

[B26] Mormann F. Fell J. Axmacher N. Weber B. Lehnertz K. Elger C. E. (2005). Phase/amplitude reset and theta-gamma interaction in the human medial temporal lobe during a continuous word recognition memory task. *Hippocampus* 15 890–900. 10.1002/hipo.20117 16114010

[B27] Novak P. Lepicovska V. Dostalek C. (1992). Periodic amplitude modulation of EEG. *Neurosci. Lett.* 136 213–215. 10.1016/0304-3940(92)90051-8 1641193

[B28] Onslow A. C. E. Bogacz R. Jones M. W. (2011). Quantifying phase-amplitude coupling in neuronal network oscillations. *Progr. Biophy. Mol. Biol.* 105 49–57. 10.1016/j.pbiomolbio.2010.09.007 20869387

[B29] Özkurt T. E. Schnitzler A. (2011). A critical note on the definition of phase-amplitude cross-frequency coupling. *J. Neurosci. Methods* 201 438–443. 10.1016/j.jneumeth.2011.08.014 21871489

[B30] Penny W. D. Duzel E. Miller K. J. Ojemann J. G. (2008). Testing for nested oscillation. *J. Neurosci. Methods* 174 50–61. 10.1016/j.jneumeth.2008.06.035 18674562 PMC2675174

[B31] Pfurtscheller G. (1976). Ultralangsame schwankungen innerhalb der rhythmischen aktivität im alpha-band und deren mögliche ursachen. *Pflugers Arch.* 367 55–66. 10.1007/BF00583657 1034286

[B32] Rasch B. Friese M. Hofmann W. Naumann E. (2006). *Quantitative Methoden 2: Einführung in die Statistik.* Berlin: Springer.

[B33] Samiee S. Baillet S. (2017). Time-resolved phase-amplitude coupling in neural oscillations. *NeuroImage* 159 270–279. 10.1016/j.neuroimage.2017.07.051 28757194

[B34] Sotero R. C. (2016). Topology, cross-frequency, and same-frequency band interactions shape the generation of phase-amplitude coupling in a neural mass model of a cortical column. *PLoS Computat. Biol.* 12:e1005180. 10.1371/journal.pcbi.1005180 27802274 PMC5089773

[B35] Tort A. B. L. Komorowski R. Eichenbaum H. Kopell N. (2010). Measuring phase-amplitude coupling between neuronal oscillations of different frequencies. *J. Neurophysiol.* 104 1195–1210. 10.1152/jn.00106.2010 20463205 PMC2941206

[B36] Tort A. B. L. Kramer M. A. Thorn C. Gibson D. J. Kubota Y. Graybiel A. M. (2008). Dynamic cross-frequency couplings of local field potential oscillations in rat striatum and hippocampus during performance of a T-maze task. *Proc. Natl. Acad. Sci. U.S.A.* 105 20517–20522. 10.1073/pnas.0810524105 19074268 PMC2629291

[B37] van der Meij R. Kahana M. Maris E. (2012). Phase-amplitude coupling in human electrocorticography is spatially distributed and phase diverse. *J. Neurosci.* 32 111–123. 10.1523/JNEUROSCI.4816-11.2012 22219274 PMC6621324

[B38] Vanhatalo S. Palva J. M. Holmes M. D. Miller J. W. Voipio J. Kaila K. (2004). Infraslow oscillations modulate excitability and interictal epileptic activity in the human cortex during sleep. *Proc. Natl. Acad. Sci. U.S.A.* 101 5053–5057. 10.1073/pnas.0305375101 15044698 PMC387372

[B39] Vinck M. van Wingerden M. Womelsdorf T. Fries P. Pennartz C. M. A. (2010). The pairwise phase consistency: A bias-free measure of rhythmic neuronal synchronization. *NeuroImage* 51 112–122. 10.1016/j.neuroimage.2010.01.073 20114076

[B40] Vosskuhl J. Huster R. J. Herrmann C. S. (2015). Increase in short-term memory capacity induced by down-regulating individual theta frequency via transcranial alternating current stimulation. *Front. Hum. Neurosci.* 9:257. 10.3389/fnhum.2015.00257 26005411 PMC4424841

[B41] Widmann A. Schröger E. Maess B. (2015). Digital filter design for electrophysiological data – a practical approach. *J. Neurosci. Methods* 250 34–46. 10.1016/j.jneumeth.2014.08.002 25128257

[B42] Zhivomirov H. (2013). Pink, red, blue and violet noise generation with matlab implementation. Available at: http://www.mathworks.com/matlabcentral/fileexchange/42919-pink--red--blue-and-violet-noise-generation-with-matlab-implementation/content/rednoise.m, (accessed 4 17),

[B43] Zhivomirov H. (2018). A method for colored noise generation. *Roman. J. Acous. Vibration* 1 14–19.

