# Supplementary material for: Quantification of Phase-Amplitude Coupling in Neuronal Oscillations: Comparison of Phase-Locking Value, Mean Vector Length, Modulation Index, and Generalized-Linear-Modeling-Cross-Frequency-Coupling
Source: Front Neurosci. 2019 Jun 7;13:573. doi: 10.3389/fnins.2019.00573 (PMC6592221; doi:10.3389/fnins.2019.00573)
Supplement: Supplementary file 1 [file Data_Sheet_1.docx]

Appendix A.

In this appendix we provide our MATLAB script for simulating data and calculating phase-amplitude coupling.

% Script for simulating EEG data for testing cross-frequency

% phase-amplitude coupling methods

% defining center frequencies, for which to calculate coupling

freq4phs = 9; % in Hz

freq4amp = 60; % in Hz

% amount of simulations (e. g. 100 for coupling or 10000 for no coupling)

nrpts = 100;

% define parameters used for modifying the simulated signal

trial_length = [400,2500,5000]; % in msec

sampling_rate = [500 1000]; % in Hz

noise = [0.9,1,1.1]; % additional noise

modulation_strength = [0.9,1,1.1];

mw_max_dur = (1000/((freq_ph_min+freq_ph_max)/2))/4;

modulation_width = [0.9*mw_max_dur,mw_max_dur,1.1*mw_max_dur]; % in ms

phasic = {'mono','bi'};

ntrial = 30; % amount of trials extracted from the simulated data

niter = 1000; % amount of iterations for permutation testing

% defining frequency band borders accoring to the center frequencies

freq_ph_min = freq4phs-1; % in Hz

freq_ph_max = freq4phs+1; % in Hz

freq_amp_min = freq4amp-freq_ph_max; % in Hz

freq_amp_max = freq4amp+freq_ph_max; % in Hz

% defining data length according to trial length and amount of trials

datalength = zeros(size(trial_length));

for n=1:length(trial_length)

datalength(n) = (trial_length(n)/1000 * ntrial) + ntrial;

end

% initialising phase-amplitude plot

nbin = 18;

winsize = 2*pi/nbin;

position = zeros(1,nbin); % in radians

for j=1:nbin

position(j) = -pi+(j-1)*winsize;

end

% initialise empty cell matrix for PAC values

PAC_values = cell(nrpts,length(datalength),length(sampling_rate), ...

length(noise),length(modulation_strength),length(modulation_width), ...

length(phasic));

% shuffling random number generator

rng('shuffle'); % creates a different seed for the rand-nr-generator

rng_settings = rng; % saves current settings of the generator

rng_example = rand(1,5); % saves 5 examples of the generated random nrs

eeglab;

cd 'C:\EEGLAB\eeglab14_1_2b\plugins\noise generation' % function for ...

% noise generation downloaded from ...

% https://www.mathworks.com/matlabcentral/fileexchange/42919-pink-red- ...

% blue-and-violet-noise-generation-with-matlab

runIdx = 1;

for iteri=1:nrpts

% simulating EEG structure for eegfilt

EEG.srate = 1000; % initial sampling rate

EEG.pnts = max(datalength)*EEG.srate;

EEG.nbchan = 1;

EEG.trials = 1;

EEG.data = rednoise(EEG.pnts);

EEG.times = 0:1/EEG.srate:max(datalength)-1/EEG.srate;

rawEEG = EEG;

for dli=1:length(datalength)

for sri=1:length(sampling_rate)

for noi=1:length(noise)

for msi=1:length(modulation_strength)

for mwi=1:length(modulation_width)

for phi=1:length(phasic)

EEG = rawEEG;

% change original data to defined length

EEG.data = EEG.data(1:datalength(dli)*EEG.srate);

EEG.pnts = length(EEG.data);

EEG.times = 0:1/EEG.srate:datalength(dli)-1/EEG.srate;

EEG.xmax = (datalength(dli)-1/EEG.srate);

% resample original data

EEG = pop_resample(EEG, sampling_rate(sri));

% initialise random time points for permuted phase time series in data

% points

numpoints_trial = (datalength(dli) - ntrial) / ntrial * sampling_rate(sri);

% bandpass filtering phase_signal

sig_ph = pop_eegfiltnew(EEG, freq_ph_min, freq_ph_max, [], 0, [], 0);

sig_ph = sig_ph.data; % lower frequency signal for phase

% bandpass filtering amplitude_signal

sig_amp = pop_eegfiltnew(EEG,freq_amp_min, freq_amp_max, [], 0, [], 0);

sig_amp = sig_amp.data; % higher frequency signal for power

% modulate amplitude signal, if there is modulation, i.e. I~=0;

if modulation_strength(msi)~=0

% creation of a Hanning window with "uneven" length, in order to be

% able to place it evenly around the extreme values

if rem(ceil((modulation_width(mwi)/1000*EEG.srate)),2)

myHanning = modulation_strength(msi) .* ...

hann(ceil(modulation_width(mwi)/1000*EEG.srate))';

else % the data length of the Hanning window is even;

% therefore one data point is subtracted

myHanning = modulation_strength(msi) .* ...

hann(ceil((modulation_width(mwi)/1000*EEG.srate)-1))';

end

modulation_width_final = length(myHanning) * 1000/EEG.srate;

% find peak indexes

IdxMaxima = findpeaks(sig_ph);

if strcmp(phasic(phi),'mono') % modulation only at peaks

% Looping through all peaks and multiply amplitude with hanning

% window

for exi=1:length(IdxMaxima.loc)

StartIdx = IdxMaxima.loc(exi)-floor(length(myHanning)/2);

EndIdx = IdxMaxima.loc(exi)+floor(length(myHanning)/2);

if StartIdx < 1

y = 1-StartIdx;

shortenedHann = myHanning(y+1:end);

StartIdx = 1;

sig_amp(1,StartIdx:EndIdx) = sig_amp(1,StartIdx:EndIdx).*(1+shortenedHann);

elseif EndIdx > length(sig_amp)

y = EndIdx - length(sig_amp);

shortenedHann = myHanning(1:end-y);

EndIdx = length(sig_amp);

sig_amp(1,StartIdx:EndIdx) = sig_amp(1,StartIdx:EndIdx).*(1+shortenedHann);

else

sig_amp(1,StartIdx:EndIdx) = sig_amp(1,StartIdx:EndIdx).*(1+myHanning);

end

end

elseif strcmp(phasic(phi),'bi') % modulation at peaks and throughs

% find peaks indexes: already done

% find through indexes

DataInv = 1.01*max(sig_ph) - sig_ph;

IdxMinima = findpeaks(DataInv);

IdxExtrms(1:length(IdxMaxima.loc)) = IdxMaxima.loc;

IdxExtrms(length ...

(IdxMaxima.loc)+1:length(IdxMaxima.loc)+length(IdxMinima.loc)) = ...

IdxMinima.loc;

% Looping through all peaks and throughs and multiply amplitude with

% Hanning window

for exi=1:length(IdxExtrms)

StartIdx = IdxExtrms(exi)-floor(length(myHanning)/2);

EndIdx = IdxExtrms(exi)+floor(length(myHanning)/2);

if StartIdx < 1

y = 1-StartIdx;

shortenedHann = myHanning(y+1:end);

StartIdx = 1;

sig_amp(1,StartIdx:EndIdx) = sig_amp(1,StartIdx:EndIdx).*(1+shortenedHann);

elseif EndIdx > length(sig_amp)

y = EndIdx - length(sig_amp);

shortenedHann = myHanning(1:end-y);

EndIdx = length(sig_amp);

sig_amp(1,StartIdx:EndIdx) = sig_amp(1,StartIdx:EndIdx).*(1+shortenedHann);

else

sig_amp(1,StartIdx:EndIdx) = sig_amp(1,StartIdx:EndIdx).*(1+myHanning);

end

end

end

else

% if there is no modulation, the above procedure

% does not need to be carried out

modulation_width_final = 0;

end

% generate additional noise

EEG.data = rednoise(EEG.pnts);

noise_ph = pop_eegfiltnew(EEG, freq_ph_min, freq_ph_max,[],0,[],0);

noise_ph = noise_ph.data;

noise_amp = pop_eegfiltnew(EEG,freq_amp_min,freq_amp_max,[],0,[],0);

noise_amp = noise_amp.data;

% final synthetic signal

sig_ph = sig_ph + noise(noi) .* noise_ph;

sig_amp = sig_amp + noise(noi) .* noise_amp;

% extract amplitude and phase from simulated continiuous data

phase_ts_cont = angle(hilbert(sig_ph));

amplt_ts_cont = abs(hilbert(sig_amp));

ph_ap_ts_cont = angle(hilbert(abs(hilbert(sig_amp))));

% epoch data (as often real data does conatin data inconsistencies)

triallength = (datalength(dli) - ntrial) / ntrial; % in sec

marker_dist = 1+triallength;

event_latency = ...

EEG.srate/2:marker_dist*EEG.srate:marker_dist*ntrial*EEG.srate;

start = 1;

phase_ts = zeros(1,triallength*EEG.srate*ntrial);

amplt_ts = zeros(1,triallength*EEG.srate*ntrial);

ph_ap_ts = zeros(1,triallength*EEG.srate*ntrial);

for eli=1:ntrial

phase_ts(1,start:eli*triallength*EEG.srate) = ...

phase_ts_cont(event_latency(eli):event_latency(eli)+ ...

(triallength*EEG.srate-1));

amplt_ts(1,start:eli*triallength*EEG.srate) = ...

amplt_ts_cont(event_latency(eli):event_latency(eli)+ ...

(triallength*EEG.srate-1));

ph_ap_ts(1,start:eli*triallength*EEG.srate) = ...

ph_ap_ts_cont(event_latency(eli):event_latency(eli)+ ...

(triallength*EEG.srate-1));

start = start + triallength*EEG.srate;

end

% calculate mean amplitude in phase bins for MI

MeanAmp = zeros(1,nbin);

for j=1:nbin

MeanAmp(j) = mean(amplt_ts(find ...

(phase_ts >= position(j) & phase_ts < position(j)+winsize)));

end

% calculate observed MI (complex-valued composite signal)

NormAmp = MeanAmp/sum(MeanAmp);

ShannonEntropy = -sum(NormAmp.*log(NormAmp));

KL_Dist = log(nbin)-ShannonEntropy;

obs_MI = KL_Dist / log(nbin);

% calculate observed MVL (complex-valued composite signal)

obs_MVL = mean(amplt_ts(:).*exp(1i*phase_ts(:)));

% calculate observed PLV (complex-valued composite signal)

obs_PLV = mean(exp(1i*(phase_ts(:) - ph_ap_ts(:))));

% calculate observed GLM

[obs_GLM,obs_GLM_CI,NrCtrlPts] = glmfun(phase_ts,amplt_ts,10,'AIC');

% The glmfun.m provided by Kramer & Eden (2013), ...

% was modified, such that it takes the phase time series and amplitude

% time series instead of the band pass filtered signal.

% split continious data into trials

phase_ts = reshape(phase_ts,triallength*EEG.srate,ntrial);

amplt_ts = reshape(amplt_ts,triallength*EEG.srate,ntrial);

ph_ap_ts = reshape(ph_ap_ts,triallength*EEG.srate,ntrial);

EEG.trials = ntrial;

% calculate surrogate values

permuted_phase = zeros(length(phase_ts),ntrial);

permuted_MI = zeros(1,niter);

permuted_MVL = zeros(1,niter);

permuted_PLV = zeros(1,niter);

permuted_GLM = zeros(1,niter);

for s=1:niter

skip = randsample ...

(round(numpoints_trial*.8),ntrial) + round(numpoints_trial*.1);

for ti=1:ntrial

permuted_phase(:,ti) = phase_ts([skip(ti):end 1:skip(ti)-1],ti);

end

MeanAmpShuffeld = zeros(1,nbin);

for j=1:nbin

MeanAmpShuffeld(j) = mean(amplt_ts(find ...

(permuted_phase>=position(j)&permuted_phase<position(j)+winsize)));

end

permuted_MI(s) = (log(nbin)- ...

(-sum((MeanAmpShuffeld/sum(MeanAmpShuffeld)) .* ...

log((MeanAmpShuffeld/sum(MeanAmpShuffeld)))))) / log(nbin);

permuted_MVL(s) = abs(mean( amplt_ts(:).*exp(1i*permuted_phase(:)) ));

permuted_PLV(s) = abs(mean(exp(1i*(permuted_phase(:) - ph_ap_ts(:)))));

[permuted_GLM(s),~,~] =glmfun(permuted_phase(:),amplt_ts(:),NrCtrlPts);

end

% calculate z-standardized PAC values

MIz = ( obs_MI - mean(permuted_MI)) / std(permuted_MI);

MVLz = (abs(obs_MVL) - mean(permuted_MVL)) / std(permuted_MVL);

PLVz = (abs(obs_PLV) - mean(permuted_PLV)) / std(permuted_PLV);

GLMz = ( obs_GLM - mean(permuted_GLM)) / std(permuted_GLM);

MVLphase = angle(obs_MVL);

PLVphase = angle(obs_PLV);

% save PAC values

parameter_name = ['freq' num2str(freq4phs) 'to' num2str(freq4amp) '_' ...

num2str(datalength(dli)) 'DL_' ...

num2str(EEG.srate) 'SR_' ...

num2str(noise(noi)*100) 'NO_' ...

num2str(modulation_strength(msi)*10) 'MS_' ...

num2str(modulation_width_final) 'MW_' char(phasic(phi))];

toSave = {parameter_name,obs_MI,MIz,obs_MVL,MVLz,MVLphase, ...

obs_PLV,PLVz,PLVphase,obs_GLM,obs_GLM_CI,NrCtrlPts,GLMz};

PAC_values{iteri,dli,sri,noi,msi,mwi,phi} = toSave;

% clear variables because of varying data length

EEG.data=[]; EEG.srate=[]; EEG.times=[]; EEG.pnts=[]; EEG.nbchan=[];

EEG.trials=[];

clear sig_amp noise_amp amplt_ts amplt_ts_cont sig_ph noise_ph

clear phase_ts phase_ts_cont permuted_phase ph_ap_ts ph_ap_ts_cont

clear MeanAmp MeanAmpShuffeld NormAmp event_latency IdxMaxima myHanning

clear bedname toSave

end

end

end

end

end

end

save('tmp_results','PAC_values','rng_settings')

end

save('results','PAC_values','rng_settings','rng_example')

Zhivomirov, H. (2013). Pink, Red, Blue and Violet Noise Generation with Matlab Implementation. Accessed April 17, 2016, http://www.mathworks.com/matlabcentral/fileexchange/42919-pink--red--blue-and-violet-noise-generation-with-matlab-implementation/content/rednoise.m

Kramer, M. A., and Eden, U. T. (2013). Assessment of cross-frequency coupling with confidence using generalized linear models. Journal of Neuroscience Methods 220, 64–74. doi: 10.1016/j.jneumeth.2013.08.006
